# Report of the “satisfaction” survey amongst public health services nurses in Port Said

**DOI:** 10.1186/s12912-021-00707-y

**Published:** 2021-10-14

**Authors:** Saverio Bellizzi, Susanna Padrini

**Affiliations:** 1 Medical Epidemiologist, Independent Consultant, Geneva, Switzerland; 2 A.I.S.P.O. Associazione Italiana per la Solidarietà tra i Popoli, Milan, Italy

**Keywords:** Nurse, Egypt, Job satisfaction, Occupational health, Quality services

## Abstract

**Background:**

There is a paucity of evidence regarding the job experience of nurses in Egypt. An unpublished previous pilot study conducted in the Port Said Technical Nursing Institute, which was based on 36 participants, showed that almost half of nurses were satisfied with their job; on the other hand, nurses indicated low salaries and high work-loads as main reasons for dissatisfaction. We explored job satisfaction of nurses working in public health services of the Port Said Governorate to inform future healthcare policy.

**Methods:**

A cross-sectional study including nurses from different public health services was conducted. Questionnaires were delivered in a sample of primary health care facilities as well as in the Port Said Governorate public hospital. Following a literature review, eight components were identified as contributors to job satisfaction; two closed questions for each of the eight components and two open questions were devised for a total of 18 questions.

**Results:**

The final study population consisted of 285 individuals. Almost 40.0% of the participants felt safe in their clinical environment while around 10.0% disagree on this. Almost 70.0% of participants complained about high work-load due to shortage of staff in the respective clinical area. Almost 85.0% of nurses reported that their salary did not cover living cost while only 13.0% indicated earning a fair salary. Almost 60.0% agreed with the fact that they have regular opportunities to develop in their career.

**Conclusion:**

Increasing job satisfaction among nurses in Egypt is critical to ensure quality of care for patients. Issues such as salary, staffing and cooperation with colleagues deserve specific attention.

## Background

Job satisfaction is a multifaceted concept that covers emotional, physical, psychological, moral and social satisfaction that individuals derive from their chosen profession [1].

In health services, and more specifically in the area of care provided by nurses, job satisfaction has an impact on patient outcome, nurse performance, retention of nursing staff and absenteeism. It also influences commitment to the organization, organizational change and the nursing profession, working environment/conditions, relationship with co-workers and managers, and other relevant factors [2–5].

Egypt, like other countries of the world, has a strong tradition of caring for the sick, and maintaining and promoting the health of its citizens. Nurses are the backbone of primary health and hospital services in Egypt and are in high demand. However, Egypt has a chronic nursing shortage. In 2015, there were 19.2 nurses and midwives for every 10,000 Egyptians [6], corresponding to around 25% of the value in UK (81.7) and around 15% of that in Australia (128.1). A 2017 report indicated the presence of 202,542 nurses in Egypt [7].

There are two routes generally followed to becoming a licensed or registered nurse in Egypt: second-level nurses typically complete a 2-year training programme that may include a 6-month internship (diploma). A qualification is issued at either certificate or diploma level. First-level nurses usually complete a 3–4-year training programme culminating in a degree-level qualification (bachelor). Second-level nurses are typically prepared in programmes governed by the vocational rather than the higher education sector. Therefore, programmes are typically delivered from vocationally orientated technical institutes, community colleges or trade schools rather than higher education formally accredited universities [8]. The majority (almost 90%) of nurses in Egypt have a certificate or diploma of nursing and only 6–8% had a certificate of bachelor of nursing [7].

We built on the previous pilot study carried out in the Port Said Technical Nursing Institute based on 36 participants, which showed almost 50% were satisfied with their salary, but were not satisfied with their workload.

Using the same research question “What is the level of job satisfaction of nurses working in public hospitals in Port Said?” we conducted a study with representation of all the public health structures of the Port Said Governorate.

## Methods

### Study design

A cross sectional study including nurses from different specialties was conducted. Questionnaires were delivered to a sample of primary health care facilities as well as in the Port Said Governorate public hospital. Health facilities were randomly selected using population proportionate to the size (PPS) of the estimated population of nurses in all the public health structures in the Governorate.

### Sample size

Given the total population of nurses in the Port Said Governorate is 1100, and assuming parameters such as a baseline proportion of 50.0% for the outcome of interest, a response rate of 90.0% of those invited to participate in the surveyed population, a power to detect a difference of 90.0%, and an alpha error of 0.05 for the total study sample, our sample size consisted of 295 participants.

### Questionnaires

Following a literature review eight components were identified as contributors to job satisfaction; two closed questions for each of the eight components and two open questions were developed making a total of 18 questions (Table 1). The questionnaire was tested for both validity and reliability. The validity was determined by measuring the content validity, construct validity, criterion validity and concurrent validity. Internal consistency reliability and test-retest reliability were also examined to estimate the stability of results. The internal consistency reliability had a Cronbach’s coefficient level of 0.91 while correlation of test-retest reliability was 0.91.

**Table 1:** Questionnaire with percentage results and 95% Confidence Interval categorized into five categories for 285 nurses in Port Said, Egypt

Questions	Strongly agree		Agree		Partly agree		Do not agree		Definitely disagree	
	%	95% CI	%	95% CI	%	95% CI	%	95% CI	%	95% CI
*Feel safe when working in my clinical environment*	8.1	5.4–11.9	39.6	34.1–45.5	10.1	7.1–14.3	32.2	27.1–38.0	9.8	6.8–13.9
*Working environment makes me feel anxious and stressed*	23.1	18.6–28.4	43.8	38.2–49.7	10.2	7.1–14.3	20.7	16.4–25.8	2.1	0.9–4.6
*In my clinical area I feel I am part of a team*	20.7	16.4–25.8	64.5	58.8–69.9	4.9	2.9–8.1	8.4	5.7–12.3	1.4	0.5–3.7
*I feel supported by the manager*	7.4	4.8–11.1	33.7	28.4–39.4	14.7	11.1–19.4	35.4	30.1–41.2	8.8	6.0–12.7
*My work load is too high because not enough nurses in my clinical area*	31.7	26.5–37.4	37.3	31.8–43.1	5.3	3.0–8.6	23.6	19.0–28.9	2.1	0.9–4.6
*I cannot give proper care because too high work load*	10.9	7.7–15.1	21.7	17.3–26.9	9.8	6.8–13.9	50.1	44.3–56.0	7.4	4.8–11.1
*My nursing salary covers my living cost*	0.7	0.1–2.8	9.1	6.3–13.1	5.6	3.4–9.0	38.6	33.1–44.4	46.0	40.2–51.8
*I believe I get a fair salary*	1.4	0.5–3.7	12.0	8.7–16.3	6.3	4.0–9.8	37.7	32.2–43.5	42.6	36.9–48.5
*I have the opportunity to express my opinion on decision about patient care*	8.1	5.4–11.9	49.1	43.3–54.9	14.7	11.1–19.4	22.8	18.3–28.1	5.3	3.2–8.6
*My supervisor gives me responsibility when appropriate*	9.5	6.6–13.5	47.4	41.6–53.2	13.3	9.8–17.8	27.7	22.8–33.2	21.0	0.9–4.6
*When I request changes to my work schedule, these can usually be accomodated*	9.8	6.9–13.9	47.5	41.7–53.4	12.3	9.0–16.7	25.3	20.6–30.8	4.9	2.9–8.2
*I have regular opportunities to develop my career*	10.2	7.1–14.2	49.1	43.3–54.9	9.1	6.3–13.1	25.3	20.5–30.7	6.3	4.0–9.8
*My job is very fulfilling*	13.3	9.8–17.8	44.6	38.8–50.4	6.3	4.0–9.8	17.2	13.2–22.0	18.6	14.5–23.6

Questionnaires was translated from the English language version into Arabic and tested in a previous pilot study in 4 hospitals in Port Said.

### Statistical analysis

A detailed description of the overall study population with stratification by health services and specialties as well as by socio-demographic variables was performed. A table reporting percentage and 95% confidence intervals for answer to each item of the questionnaire was produced.

Number and proportion of all answers to the two open questions were computed.

## Results

The final study population consisted of 285 individuals after 10 (10/295 = 3.4%) nurses declined to participate at the survey. Thirty-six nurses were from Al Zahouer Hospital, 11 from El Masah el Bahary Hospital, 35 from El Naser Hospital, 16 from the Epidemic Hospital, 12 from the Ophtalmology Hospital, 32 from Port Fouad Hospital, 58 from Port Said General Hospital, and 85 from Primary Health Centres.

In terms of nursing specialties, 31 participants worked in the intensive care unit (ICU), 5 were part of teams in the Infection Prevention and Control unit, 46 worked in the inpatient ward, 12 in the pediatric ward, 6 in the blood bank, 29 in the emergency room (ER), 11 in the operation theatre, 105 in outpatient departments, and 40 from other areas such as sterilization and radiology.

The majority of the sample were females (*N* = 274; 96.1%) and most of participants were married (*N* = 258; 90.5%). Most of survey participants were staff nurses (*N* = 241; 84.6%), followed by head nurses (*N* = 19;6.7%); supervisors, matrons and others represented less than 5.0% of the total study population.

Around one third of participants had been working for more than 20 years (*N* = 102), one fifth had been working for between 10 and 14 years (*N* = 61), one fifth between 15 and 20 years (*N* = 59), and the remaining proportion for less than 10 years (*N* = 63); only 26 nurses had been working for less than 5 years. Almost 40.0% of the participants felt safe in their working environment while around 10.0% completely disagree on this view. Two thirds of participants agreed that their working environment made them feel anxious and stressed. The distribution of nurses who felt supported by their manager was balanced, with around 40% in agreement, 40% in disagreement, and 20% partly agreeing on the subject. Almost 70.0% of participants reported staff shortages in their respective clinical area as reason for high workload, while almost 60.0% disagreed that this meant they were not able to give proper care because of their high workload. Almost 85.0% of the nurses reported that their salary did not cover their living cost while only 13.0% agreed that they earned a fair salary.

Around 55.0% of participants had the opportunity to express their opinion on patient care with medical staff (15% partly agreed on this, and 28% disagreed). A very similar pattern was found for the questions *“my supervisor gives me responsibility when appropriate”* and *“when I request changes to my work schedule, these can usually be accommodated”* (55% agree, 15% partly agree, and the remaining participants were in disagreement). Almost 60.0% agreed they had regular opportunities to develop in their career. Slightly more than 60% found their job fulfilling, while more than 35% disagreed on this aspect.

When asked on what they enjoyed most about their job, 142 (56.1%) nurses replied *“helping others*”, which mainly included curing/healing patients; 64 (25.0%) indicated “*cooperation with colleagues*” while a minority reported “*love for their job*” (7%), “*gratitude from patients*” (5%) and “*others*”. Lack of gratitude and the attitude of patients was the most cited answer for what they enjoyed the least (*N* = 49; 23%), followed by the lack of organization at work (*N* = 45; 21%), no reward from manager (*N* = 35;17%), bad cooperation with colleagues (*N* = 28; 13%), and low salary (*N* = 14; 6.7%).

## Discussion

As mentioned by Whitman et al., understanding how to improve job satisfaction as an outcome is important as it relates to employee health, productivity, and job performance [9]. Nursing satisfaction is a global well-recognized issue because of its direct consequences on the quality and safety of patient care services [4].

This small study highlighted how nurses in Port Said Governorate embark on their profession with the spirit of “helping others”. However, critical aspects are likely to undermine this spirit of caring. Lack of organization, few rewards from managers, poor cooperation with colleagues and especially low salaries may cause stress and anxiety, which in turn hampers good performances.

A recent report from Mansoura University Hospital in Egypt has revealed an overall high prevalence of nurses encountering low job satisfaction (61.8%) [10]. Lack of communication between colleagues and supervisors, and low support at work predicted this poor outcome variable. The high prevalence of low job satisfaction among public nurses was explained in reports from other low- and middle-income countries where nurses working in the public sector usually work under poor conditions and may have access to limited resources [11]. Also, aspects like personal, social, and work features may affect job satisfaction [12]. These features may either buffer or exacerbate negative aspects such as low salary and poor working conditions. It is therefore crucial to inspect the sociodemographic and work characteristics that could be associated with low job satisfaction of the studied nurses.

Several studies have reported that higher rates of nurse satisfaction is associated with better quality and safety of care for patients [13–15]. While sufficient development of professional identity can contribute to individual autonomy, organizational behaviour is another important contributor to satisfaction [16]. Previous study conducted on Ethiopian nurses working in public hospital, reported that absence of mutual understanding at work was a significant predictor of low job satisfaction which was in accordance with our result. The Ethiopian study found also other similar predictors as excess work and lack of organization [17]. Jayasuriya et al. on the other hand concluded that work climate, supervisory support, community support were significant predictor of job satisfaction [18].

To our knowledge, there is a dearth of evidence on the relationship between nurses’ job satisfaction and in-job and external characteristics (socio-demographic) particularly in countries suffering from nursing shortage like Egypt. Addressing specific issues such as low-salaries, attitude of supervisors towards positive relation with staff nurses and organization of work will be crucial to increase job satisfaction and consequently improve the quality of healthcare.

## Conclusions

This study contributes to provide evidence on key aspects that affect job satisfaction among nurses in settings with shortage of human resources like Egypt. Findings are critical to ensure quality of care for patients and should carefully considered by policymakers.

## Data Availability

All data analysed in this study are available under request.
